# Hospital-related incidents; causes and its impact on disaster preparedness and prehospital organisations

**DOI:** 10.1186/1757-7241-17-26

**Published:** 2009-06-03

**Authors:** Amir Khorram-Manesh, Annika Hedelin, Per Örtenwall

**Affiliations:** 1 Prehospital and Disaster Medicine Centre, Gothenburg, Sweden

## Abstract

**Background:**

A hospital's capacity and preparedness is one of the important parts of disaster planning. Hospital-related incidents, a new phenomenon in Swedish healthcare, may lead to ambulance diversions, increased waiting time at emergency departments and treatment delay along with deterioration of disaster management and surge capacity. We aimed to identify the causes and impacts of hospital-related incidents in Region Västra Götaland (western region of Sweden).

**Methods:**

The regional registry at the Prehospital and Disaster Medicine Center was reviewed (2006–2008). The number of hospital-related incidents and its causes were analyzed.

**Results:**

There were an increasing number of hospital-related incidents mainly caused by emergency department's overcrowdings, the lack of beds at ordinary wards and/or intensive care units and technical problems at the radiology departments. These incidents resulted in ambulance diversions and reduced the prehospital capacity as well as endangering the patient safety.

**Conclusion:**

Besides emergency department overcrowdings, ambulance diversions, endangering patient s safety and increasing risk for in-hospital mortality, hospital-related incidents reduces and limits the regional preparedness by minimizing the surge capacity. In order to prevent a future irreversible disaster, this problem should be avoided and addressed properly by further regional studies.

## Background

Region Västra Götaland is the public healthcare provider for the western part of Sweden, with a population around 1.5 million. This task is achieved through around 150 primary healthcare centres and 10 emergency hospitals (the largest Sahlgrenska University Hospital in Gothenburg). PKMC (Prehospital Disaster and Medicine Centre) is a regional unit responsible for (medical) risk assessment and emergency planning as well as staff training in disaster management. The center is also associated with Sahlgrenska Academy in disaster research funded by the National Board of Health and Welfare. PKMC assumes command and control on a regional ("gold") level in case of major incidents/disasters [1]. All incidents and consequent activities caused by them have been registered at the centers registry (PKMC registry) since 1999 and can be analyzed retrospectively.

The current economical crisis within most healthcare systems has resulted in local, regional and national plans to reduce economic deficits. Most of these plans aim to increase the healthcare systems effectiveness by reducing hospital beds and expanding out-patients departments, which in turn challenges the mode of operation at emergency departments (ED) [2,3]. During past decades the increasing number of patients at ED s treatment areas has resulted in a work overload, making EDs to operate beyond their capacity. Hospital bed shortage enforces a rapid turnover of patients, increasing the number of patients discharged as early as possible and endangers patient safety. Together with increasing number of non-urgent patients these are main factors causing ED-overcrowding [1,4,5]. To counter with ED-overcrowding escalation, new methods (e.g.triage) have been deployed, which mainly deal with its infrastructure and internal organization [1,6]. ED is, however, the hospital's main contact point with surrounding world and an important part of disaster preparedness in the area served by the hospital. An overloaded ED has a great impact on other adjacent activities e.g.prehospital organization, ambulance transports, elective production (surgery), and individual patient's safety [7-9].

During the last years a new category of incidents, "hospital-related incidents", has appeared in the PKMC registry. We hypothesized that these incidents are, directly or indirectly, associated with hospital bed shortage and ED-overcrowding and consume huge regional resources. The aim of this paper was to identify the causes of these incidents in the Region Västra Götaland (western region of Sweden) by reviewing the PKMC registry data collected between January 1^st^, 2006 and December 31^st^, 2008.

## Methods

All incoming data concerning hospital-related incidents in Region Västra Götaland, between 1^st ^of January 2006 and 31^st ^of December 2008, was collected. An incident was defined as an alert from EMS (emergency medical services) dispatch centre to the regional duty officer on call (RTiB). The RTiBs have medical background as within emergency care and special training in management of major incidents. They have a thorough understanding of regional resources. As a second line senior consultants (RBL) also with special training in major incident management are available on a 24/7 basis.

Every time a contact is taken between SOS Alarm and the RTiB, data concerning this incident and the actions that resulted are entered into a web-based registry (Saltwater™) [10]. These data were reviewed and analyzed for a 3-year period. The data concerning hospital-related incidents and their impacts on that hospital's or adjacent hospital's ordinary activities were extracted and evaluated. The causes of subnormal capacity at affected hospitals were then divided into following groups; hospital bed shortage (no details specified), bed shortage at intensive care unit, bed shortage at ordinary wards, emergency departments overcrowding and technical dysfunction at radiology departments.

## Results

**Figure 1:**
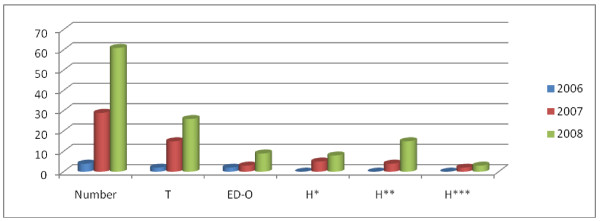
**Shows the number and causes of healthcare related incidents for year 2006 until 2008 in diagram**. T: Technical dysfunction at radiology department. ED-O: Emergency Department Overcrowding. H*: Hospital Bed Shortage Ordinary Wards. H**: Hospital Bed Shortage Intensive Care Units. H***: Hospital Bed Shortage Intensive Care Units Respirator.

There were an increasing number of hospital-related incidents between 2006-01-01 and 2008-12-31, leading to ED-overcrowding and ambulance diversions. Four incidents were registered in 2006, of which one was due to bed shortage at intensive care units, one bed shortage at ordinary ward and two due to technical dysfunction at a radiology department. In these occasions ordinary patients were referred to other hospitals directly from ED, while critically ill patients already admitted or on their way to the ED by ambulances, were transported to other hospitals. The number of incidents has then steadily increased during 2007 and 2008, reaching its peak at 61 incidents in 2008, which is fifteen times higher than that in 2006 (Table 1, Figure 1). During the same period, besides 61 healthcare related missions, 1046 other regional (e.g.traffic accidents, sport events), national (e.g.storm, flooding), and international (e.g.terrorist actions, evacuation of Swedish citizens from war zones), incidents were also entered into the PKMC registry.

**Table 1:** Shows the number and the causes of incidents from 1^st ^of January 2006 until 31^st ^of December 2008.

Year	Number	T	ED-O	H*	H**	H***
2006	4	2	2	0	0	0

2007	29	15	3	5	4	2

2008	61	26	9	8	15	3

T: Technical dysfunction at radiology department

ED-O: Emergency Department Overcrowding

H*: Hospital Bed Shortage Ordinary Wards

H**: Hospital Bed Shortage Intensive Care Units

H***: Hospital Bed Shortage Intensive Care Units Respirator

Bed shortage at intensive care units could either be due to high inflow of operated patients or high admission of critically ill patients (in need of assisted ventilation). The higher rate of operated patients was directly related to higher number of planned operations and simultaneous increasing in number of emergency cases. These numbers changed in 2008 to 35% and 65% for bed and respirators shortage, respectively. The lack of hospital beds at ordinary wards was mainly due to overloaded wards. The impact on individual patients or patients groups or the number of patients affected could not be assessed by analyzing available data. These incidents, however, led to a total number of 350 actions undertaken by the center. The actions undertaken were consultative, informative and supportive. However, if necessary, the centre intervenes to coordinate and redistribute regional resources by contacting hospitals, emergency departments, and prehospital organizations. Consequently, unaffected patients and healthy individuals were advised to visit their general practitioners, healthcare centers or other hospitals. Current ambulance transports were diverted and planned transports were directed to other hospitals. There was no information about the severity of diseases in transported patients; however, critically ill patients were transported to the nearest hospitals. The active time spent for coordinating and resolving these incidents was 21188 min (354 hrs or 45 working days á 8 h). The active reporting and writing time was 487 min.

**Figure 2:**
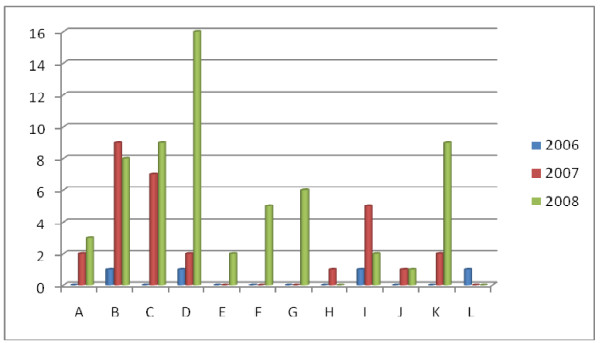
**Shows the number of incidents per year and hospitals A-L**. Hospital D represents the smallest hospital among five regional hospitals with 24 h emergency departments.

In order to find out if hospital-related incidents are hospital-dependent, the number of incidents per hospital was calculated. In 2006, only three hospitals in the Region Västra Götaland, with various sizes and capacities, were involved. However the number of hospitals reporting such incidents in the region increased during 2007 and 2008; with the highest increase in number of incidents in the smaller hospitals. Among hospitals with 24 h emergency departments, the smallest hospital (hospital D) was the one affected most (Table 2, Figure 2).

**Table 2:** Shows the number of incidents/causes for each hospitals and each year

Year	Causes	A	B	C	D	E	F	G	H	I	J	K	L
2006 Total		**0**	**1**	**0**	**1**	**0**	**0**	**0**	**0**	**1**	**0**	**0**	**1**

	T				**1**					**1**			

	ED-O		**1**										**1**

	H*												

	H**												

													

2007 Total		**2**	**9**	**7**	**2**	**0**	**0**	**0**	**1**	**5**	**1**	**2**	**0**

	T		**6**	**4**					**1**	**1**	**1**	**2**	

	ED-O	**1**		**1**	**1**								

	H*	**1**	**2**							**2**			

	H**		**1**	**2**	**1**					**2**			

													

2008 Total		**3**	**8**	**9**	**16**	**2**	**5**	**6**	**0**	**2**	**1**	**9**	**0**

	T	**1**	**7**	**5**	**1**		**5**				**1**	**6**	

	ED-O	**2**	**1**	**3**		**2**		**1**					

	H*				**2**			**5**		**1**			

	H**			**1**	**13**					**1**		**3**	

Hospitals; A, B, C, D, E, F, G, H, I, J, K, L T:

Technical dysfunction at radiology department

ED-O: Emergency Department Overcrowding

H*: Hospital Bed Shortage Ordinary Wards

H**: Hospital Bed Shortage Intensive Care Units

## Discussion

The purpose of this work was to analyze the causes of the increasing number of hospital-related incidents in Region Västra Götaland of Sweden and their impacts on the prehospital and hospital preparedness in case of major incidents. In our study the alert is initiated by the affected ED requesting the EMS dispatch centre to divert patients transported by ambulance to other EDs. One limitation to this study is the lack of possibility to measure the impact of ambulance diversions on individual patients or patients groups. The main cause of hospital-related incidents in this report was labeled as ED-overcrowding. There is however no universal definition for ED-overcrowding, as each hospital might have its own definition.

Disasters seldom occur, but if they strike a fast and effective response from healthcare services is expected. An increasing number of reports on incidents when emergency hospitals, for different reasons, cannot operate at their normal capacity are a matter of concern for patient safety as well as disaster response preparedness [7]. In the available literature hospital bed shortage and ED downsizing are reported to be some of the causes of ED-overcrowding leading to impaired responsiveness and ambulance diversions [4,6,8,9,11,12]. In our study, we could also show that hospital bed shortage and technical dysfunction at radiology departments, beside the increasing number of patients at EDs are the main reasons for ED-overcrowding in our region. Our findings (Table 1, Figure 1) are consistent with those earlier reported. Like in many other parts of the world, reduction of hospital beds and corresponding staff in combination with increasing number of out-patients treatments and coordination of activities between nearly located hospitals, have been some of the solutions to handle the economical constrain on the healthcare systems [9,11,13]. The mean length of hospital stay (LOS) has been reduced in Sweden, as well as other Scandinavian countries, having the lowest LOS worldwide [5,14]. Although these measures all seem to be logical steps taken to improve healthcare effectiveness and reducing the costs, they also, in a negative way, affect the surge capacity of a hospital. Such capacity in hospitals is necessary for proper management of extraordinary incidents and is influenced by 3 essential elements; staff, supplies/equipment, and structure [15,16]. Structure refers to both location for patients and the organizational infrastructure. A key to a successful major incidents response of a hospital is an ED that is able to effectively sort (triage) the casualties, continue or start lifesaving treatment and rapidly transfer patients to facilities for definitive treatment within the hospital. If this key function is overcrowded already at the onset of a disaster response, the outcome for the patients will be suboptimal. It is already reported that ED-overcrowding is associated with both space and staff shortage [4,7,17,18].

Hospital bed occupancy of ≥ 90% has been shown to correlate with a blocked access to the wards, defined as patients waiting in the ED for more than 8 h when the decision has been made to admit them [4,19-21]. For severely ill patients this consequently leads to initiation of extra measures e.g.multiple testing, interventions and administration of drugs during their prolonged stay in the ED [4,7,19,20]. In such situations the ED serves as a holding area for admitted patients, sometimes remaining for more than 24 h, due to the lack of beds [4]. This even includes patients in need of beds at the intensive care units. Earlier reports indicate that the average waiting time for an inpatient acute or critical care bed in the USA EDs has nearly been doubled (> 6 h) in hospital with consistently overcrowded ED. The results, besides missed diagnoses, poor outcomes, prolonged pain and suffering for some patients, long waiting times, patient dissatisfaction, more ambulance diversions, lower physician and staff productivity and higher levels of frustration among medical staff, are higher hospital costs and longer LOS [2,11-13,21].

In addition many patients in the early time period of their diseases may leave ED due to long waiting time, without treatment. Curable disease may then become more critical and incurable when they return [4]. Delay of > 6 h in bringing ED patients in critical condition to intensive care unit has also shown to increase hospital LOS and result in higher intensive care unit and hospital mortality [20].

Long-lasting hospital closure are associated with significant but temporary increase in ambulance diversions to the nearest ED. Fewer EDs and increasing number of patient visits over time, may also cause ED-overcrowding and consequent ambulance diversions [9,22]. Ambulance diversion has a huge impact on public health, since it may place the patient at risk for poor outcome, prolonged pain and suffering. Ambulance diversion results in increasing transport time between hospitals, delayed treatments and may also increase mortality in severely injured trauma patients [7,9,22]. It also results in significant loss of hospital revenue due to the throughput delays that prevent the use of existing bed capacity for additional patient admissions [23]

In conclusion hospital-related incidents are by no means extraordinary incidents, but part of the ordinary shortcoming of the healthcare system caused, among others, by reduction in number of hospital beds, downsizing and/or closure of hospitals EDs. Such measures results in overcrowding of EDs and ambulance diversions. They also endanger patient's safety and may increase in-hospital mortality. It counteracts medical preparedness by minimizing the surge capacity. In the context of disaster preparedness this problem must be further studied and properly addressed by our political decision makers [24].

## Competing interests

The authors declare that they have no competing interests.

## Authors' contributions

AK conceived and designed the study. AK, AH and PÖ performed the data analysis. AK drafted the manuscript. All authors interpreted data and critically revised the manuscript. All authors have read and approved the final manuscript
